# Minimally invasive 'step-up approach' versus maximal necrosectomy in patients with acute necrotising pancreatitis (PANTER trial): design and rationale of a randomised controlled multicenter trial [ISRCTN38327949]

**DOI:** 10.1186/1471-2482-6-6

**Published:** 2006-04-11

**Authors:** Marc GH Besselink, Hjalmar C van Santvoort, Vincent B Nieuwenhuijs, Marja A Boermeester, Thomas L Bollen, Erik Buskens, Cornelis HC Dejong, Casper HJ van Eijck, Harry van Goor, Sijbrand S Hofker, Johan S Lameris, Maarten S van Leeuwen, Rutger J Ploeg, Bert van Ramshorst, Alexander FM Schaapherder, Miguel A Cuesta, Esther CJ Consten, Dirk J Gouma, Erwin van der Harst, Eric J Hesselink, Lex PJ Houdijk, Tom M Karsten, Cees JHM van Laarhoven, Jean-Pierre EN Pierie, Camiel Rosman, Ernst Jan Spillenaar Bilgen, Robin Timmer, Ingeborg van der Tweel, Ralph J de Wit, Ben JM Witteman, Hein G Gooszen, members of the Dutch Acute Pancreatitis Study Group

**Affiliations:** 1 Department of Surgery, University Medical Center Utrecht, The Netherlands; 2 Department of Surgery, Academic Medical Center Amsterdam, The Netherlands; 3 Department of Radiology, St. Antonius Hospital Nieuwegein, The Netherlands; 4 Julius Center for Health Sciences and Primary Care, University Medical Center Utrecht, The Netherlands; 5 Department of Surgery, University Hospital Maastricht and NUTRIM institute, The Netherlands; 6 Department of Surgery, Erasmus Medical Center Rotterdam, The Netherlands; 7 Department of Surgery, Radboud University Nijmegen Medical Centre, The Netherlands; 8 Department of Surgery, University Medical Center Groningen, The Netherlands; 9 Department of Radiology, Academic Medical Center Amsterdam, The Netherlands; 10 Department of Surgery, St. Antonius Hospital Nieuwegein, The Netherlands; 11 Department of Surgery, Leiden University Medical Center, The Netherlands; 12 Department of Surgery, VU Medical Center Amsterdam, The Netherlands; 13 Department of Surgery, Meander Medical Center Amersfoort, The Netherlands; 14 Department of Surgery, Medical Center Rijnmond Zuid Rotterdam, The Netherlands; 15 Department of Surgery, Gelre Hospitals Apeldoorn, The Netherlands; 16 Department of Surgery, Medical Center Alkmaar, The Netherlands; 17 Department of Surgery, Reinier de Graaf Group Delft, The Netherlands; 18 Department of Surgery, St. Elisabeth Hospital Tilburg, The Netherlands; 19 Department of Surgery, Medical Center Leeuwarden, The Netherlands; 20 Department of Surgery, Canisius Wilhelmina Hospital Nijmegen, The Netherlands; 21 Department of Surgery, Rijnstate Hospital Arnhem, The Netherlands; 22 Department of Gastroenterology, St. Antonius Hospital Nieuwegein, The Netherlands; 23 Utrecht University, Centre for Biostatistics, The Netherlands; 24 Department of Surgery, Medical Spectrum Twente, Enschede, The Netherlands; 25 Department of Gastroenterology and Hepatology, Gelderse Vallei Ede, The Netherlands

## Abstract

**Background:**

The initial treatment of acute necrotizing pancreatitis is conservative. Intervention is indicated in patients with (suspected) infected necrotizing pancreatitis. In the Netherlands, the standard intervention is necrosectomy by laparotomy followed by continuous postoperative lavage (CPL). In recent years several minimally invasive strategies have been introduced. So far, these strategies have never been compared in a randomised controlled trial. The PANTER study (PAncreatitis, Necrosectomy versus sTEp up appRoach) was conceived to yield the evidence needed for a considered policy decision.

**Methods/design:**

88 patients with (suspected) infected necrotizing pancreatitis will be randomly allocated to either group A) minimally invasive 'step-up approach' starting with drainage followed, if necessary, by videoscopic assisted retroperitoneal debridement (VARD) or group B) maximal necrosectomy by laparotomy. Both procedures are followed by CPL. Patients will be recruited from 20 hospitals, including all Dutch university medical centres, over a 3-year period. The primary endpoint is the proportion of patients suffering from postoperative major morbidity and mortality. Secondary endpoints are complications, new onset sepsis, length of hospital and intensive care stay, quality of life and total (direct and indirect) costs. To demonstrate that the 'step-up approach' can reduce the major morbidity and mortality rate from 45 to 16%, with 80% power at 5% alpha, a total sample size of 88 patients was calculated.

**Discussion:**

The PANTER-study is a randomised controlled trial that will provide evidence on the merits of a minimally invasive 'step-up approach' in patients with (suspected) infected necrotizing pancreatitis.

## Background

The initial treatment of acute necrotizing pancreatitis is conservative [1-4]. Once (peri-)pancreatic necrosis becomes infected mortality increases steeply [3,4]. Intervention is indicated when infection of (peri-)pancreatic necrosis is proven by fine needle aspiration (FNA), when (peri-)pancreatic air collections in the necrotic cavity are depicted on computer tomography (CT) scan or when sepsis persists despite maximal support on the intensive care unit. Surgical intervention within the first 14 days after the onset of symptoms should be averted because of notoriously poor outcome in this phase of disease [4,5]. Organ failure needing intensive care treatment during the first two weeks should be interpreted as a complication of a systemic inflammatory response syndrome (SIRS).

There is no consensus in the literature on the optimal surgical strategy in infected necrotizing pancreatitis. In a recent systematic review we demonstrated that necrosectomy by laparotomy was accompanied by high mortality rates (15–27%) and considerable morbidity [6].

In recent years radiologists, gastrointestinal surgeons and gastroenterologists have adopted minimally invasive strategies in infected necrotizing pancreatitis [7]. Initially only practiced in patients unfit for laparotomy, but in recent years indications seem to have expanded [8-11]. Percutaneous catheter drainage (PCD)  [12-14]. endoscopic transgastric procedures [15,16]. and minimally invasive necrosectomy [9-11]. have been proposed as alternatives for necrosectomy by laparotomy. Although preliminary results are promising, current series are small, poorly comparable and a selection bias may have influenced the results [7].

It has been reported that PCD obviates the need for surgical intervention in infected necrotizing pancreatitis in 30 to 100 per cent of cases  [12-14]. In these series committed radiologists repeatedly performed repeated drainage procedures using large bore catheters. It has been argued that because of the need for repeated procedures PCD can not easily be implemented in clinical practice [17]. However, we hypothesize that 'simple' drainage with regular bore (12–14 French) percutaneous catheters can also be beneficial to the patient. Drainage of 'infected fluid under pressure' may help the patient in dealing with the (peri-)pancreatic necrosis and delay or even obviate surgical intervention in a relevant proportion of patients.

As a prelude to the present study a retrospective multicenter study was performed in 11 hospitals in the Netherlands [8]. The outcome of the different interventional strategies in (infected) necrotizing pancreatitis was assessed. The strategy most often used was laparotomy with continuous postoperative lavage (CPL) (53/106 patients) [8]. Of the patients treated with minimally invasive surgery (n = 18), two-thirds had PCD prior to surgical intervention. During surgery the percutaneous drain was used as a 'guide wire' to facilitate retroperitoneal access to the infected collections. The results of minimally invasive surgery were favourable: 11% mortality as compared to 25% after laparotomy and CPL. However, since selection bias may have played a role in the favourable results a randomised controlled trial is warranted in order to define evidence-based surgery in infected necrotizing pancreatitis.

We anticipate that a minimally invasive 'step-up approach' results in a reduction in postoperative major morbidity and mortality. The PANTER trial is designed to compare a minimally invasive 'step-up approach'with a primary maximal necrosectomyby laparotomy. The 'step-up approach' consists of percutaneous or transgastric drainage when necessary followed by minimally invasive necrosectomy.

## Methods/design

### Study objectives

To test the hypothesis that a minimally invasive 'step-up approach' will lead to a reduction of postoperative major morbidity and mortality in patients with infected (peri-)pancreatic necrosis.

### Primary endpoint

The primary endpoint is the proportion of patients with major morbidity or mortality, see Tables 1 and 2. Complications occurring subsequent to the first intervention after randomisation until three months after discharge from the hospital are compared.

### Secondary endpoints

Secondary endpoints are 'minor' complications (such as pancreatic fistula, pancreatic pseudocyst requiring intervention, pancreatic abscess, biliary strictures, incisional hernia requiring re-intervention and pancreatic insufficiency), new onset sepsis, new onset SIRS, total number of interventions, hospital and intensive care stay, quality of life and total (direct and indirect) costs.

### Definitions

The definitions of the Atlanta classification are used. *Pancreatic necrosis*: focal area's of non-enhancing pancreatic parenchyma on contrast-enhanced computer tomography (CECT). *Infected necrotizing pancreatitis*: a positive culture of pancreatic or peripancreatic necrosis obtained by FNA or the presence of air in the collections on CECT. *Suspected infected necrotizing pancreatitis*: persisting sepsis or progressive clinical deterioration despite maximal support on the intensive care unit in case of pancreatic and/or peripancreatic necrosis.

### Design of study

PANTER is a randomised controlled parallel group superiority multicenter trial.

### Participating centres

Twenty hospitals of the Dutch Acute Pancreatitis Study Group, including all Dutch university medical centres, will enrol patients (see Appendix).

### Pre-randomisation treatment protocol

All patients with acute pancreatitis will be treated by a pre-established treatment protocol consisting of enteral nutrition via a nasojejunal tube and an early endoscopic retrograde cholangiopancreatography (ERCP) with sphincterotomy in case of predicted severe biliary pancreatitis with or without obstructive cholangitis. No antibiotic prophylaxis will be administered. CECT will be performed in patients who fail to show clinical improvement after the first 7 days of hospital admission. Since many patients are referred from other centres, violation of the pre-randomisation treatment protocol is not an exclusion criteria.

#### Registration

From all patients with (peri-)pancreatic necrosis, including those who are not randomised and treated conservatively, written informed consent will be obtained for prospective registration of age, gender, onset of symptoms, interventional procedures, hospital stay, intensive care stay and mortality (alternatively consent by proxy will be obtained for patients who are unable to give consent eg. intubated patients).

#### Timing of intervention

Whenever possible it is attempted to delay surgical intervention. Ideally, the first intervention would be performed at least 30 days after onset of symptoms. Earlier intervention may be warranted in case of a rapidly deteriorating clinical condition. When infection is proven within the first 14 days, intervention may be postponed. Intervention within 14 days is only indicated in emergencies such as bowel perforation, abdominal compartment syndrome or acute bleeding. Patients with these complications are not eligible for randomisation.

### Eligibility criteria

#### Inclusion criteria

▪ age equal to or above 18 years

▪ pancreatic necrosis or peripancreatic necrosis detected on CECT.

▪ patients in whom a decision for surgical intervention has been made because of (suspected) infected (peri-)pancreatic necrosis

▪ possibility of placing a drain (either percutaneous or endoscopic) in the collection(s)

▪ written informed consent

#### Exclusion criteria

▪ previous drainage or surgical necrosectomy for (suspected) infected pancreatic necrosis, including procedures performed in referring hospitals. ERCP with or without papillotomy is allowed.

▪ previous exploratory laparotomy for acute abdomen and diagnosis of pancreatitis during laparotomy

▪ acute flare-up of chronic pancreatitis

▪ bleeding, abdominal compartment syndrome or perforation of a visceral organ as indication for intervention

▪ post-abdominal surgery necrotizing pancreatitis

Rationale: previous drainage procedures or laparotomy make it impossible to study the isolated effect of drainage of 'infected fluid under pressure'. There is essentially no indication for necrosectomy in acute fluid collections, pancreatic abscesses or pseudocysts as these collections do not contain pancreatic necrosis or necrotic debris according to the Atlanta Classification. When intervention is indicated in these types of collections this is performed by drainage procedures, although some pseudocysts may require surgical intervention [4]. Patients with chronic pancreatitis have an underlying disease with a course very different from acute pancreatitis. Patients with a second, third or fourth attack of acute pancreatitis are eligible for randomisation as long as there are no signs of chronic pancreatitis (calcification and/or pancreatic duct abnormalities).

### Randomisation

**Figure 1:**
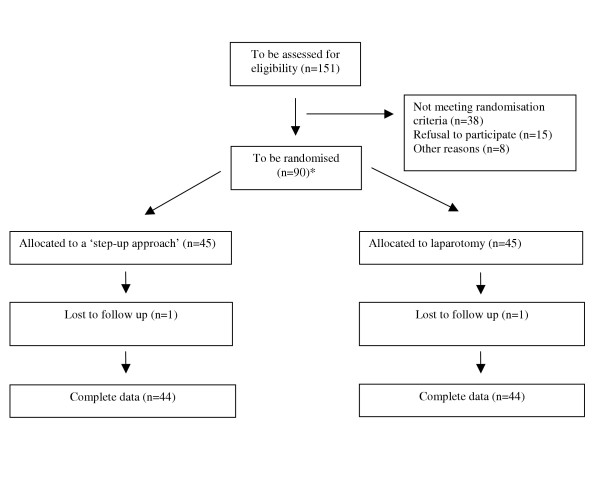
PANTER according to CONSORT.

Patients will be randomly assigned to group A ('step-up approach') or group B (laparotomy) as shown in the flowchart (see figure 1). Randomisation is performed by an Internet randomisation module (Julius Center for primary care and health sciences, UMC Utrecht, the Netherlands). Block-randomisation is used and the randomisation is stratified according to the (im-)possibility of placing a percutaneous drain through the (preferably left) retroperitoneum, since this step is essential to perform minimally invasive necrosectomy.

### Ethics

This study is conducted in accordance with the principles of the Declaration of Helsinki and 'good clinical practice' guidelines. The independent medical ethics committees of all 20 participating hospitals have approved the study protocol. Prior to randomisation, written informed consent will be obtained from all patients (alternatively consent by proxy will be obtained for patients who are unable to give consent, e.g., intubated patients).

### Safety and quality control

The indication and timing of intervention in necrotizing pancreatitis can be difficult. Therefore, prior to randomisation using CECT images and a 'summarized case report' an expert panel consisting of three surgeons (MAB, HSH, HGG), a gastroenterologist (RT) and three radiologists (TLB, MSVL, JSL) will assess the indication and feasibility of surgical, gastroenterological or radiological intervention. All procedures will be performed by experienced radiologists, gastroenterologists or gastrointestinal surgeons. All endo-/videoscopic procedures will be videotaped. In case of a planned intervention, surgeons or radiologists from (neighbouring) participating hospitals and the trial coordinator will join the procedure in order to increase experience and enhance protocol-compliance. Every four months a study group meeting will be organised in which the CECT images and the 'summarized case report' of all newly randomised patients will be discussed. An independent monitoring-committee, consisting of two surgeons, a gastroenterologist, an epidemiologist and a radiologist, will discuss (serious) adverse events and give advice to the trial steering committee.

### Statistical analysis

#### Intention-to-treat

The analysis will be performed in accordance with intention-to-treat (ITT) principle

#### Sample size and sequential interim-analysis

Sequential analysis is used to determine the difference in treatment effect on the primary endpoint (see Methods/design section), therefore no fixed sample size estimate can be given [18]. Based on the results of the Dutch retrospective multicentre audit (mortality 25% versus 11%) and PCD results from the literature is anticipated that the minimally invasive 'step-up approach' will reduce the occurrence of the primary endpoint from 45% to 16%. The expected reduction of mortality and morbidity are 10% and 19% respectively. With 80% power at 5% alpha (two-sided) complete data from about 52–77 patients will be necessary to demonstrate this effect, if it truly exists. If no such effect appears, the trial will continue until 88 patients are randomised and available for analysis as 'conventional' sample size calculation with 80% power at 5% alpha (two-sided) would require 88 patients.

Descriptive methods will be used to assess the quality of the data, comparability of treatment groups and endpoints. Continuous sequential analysis will be performed with PEST (PEST 4: user manual. MPS Research Unit (2000), the University of Reading) according to the restricted procedure as described by Whitehead [18]. Every time an endpoint occurs, data management will send a blinded, updated dataset of all included patients to the biostatistician. If one of the boundaries of the sequential analysis plot is crossed during the analysis of the cumulative data, i.e. the difference in treatment is of at least the expected magnitude, the trial steering committee will be informed and will be advised to stop randomising new patients for the trial. The trial steering committee will not be advised to stop early when no relevant differences between the treatments are observed. Next to the primary analysis, the outcome of both groups will also be adjusted forimbalance in presence of preoperative (multi-) organ failure and timing of intervention (after 28 days). These factors, including possibility of performing VARD and the individual components of the primary endpoint will also be included in a multivariate and subgroupanalysis.

#### Feasibility

The recent retrospective survey showed that in a 3-year period 106 patients had undergone surgery for necrotizing pancreatitis in 11 of the 20 participating Dutch hospitals [8]. It is expected that up to 25% of eligible patients will be excluded mainly because of interventions (percutaneous drainage or surgery) being performed prior to randomisation in referring centres. Taking further into account refusal of informed consent (10%), other reasons for drop-out (5%) and loss-to-follow-up (2.5%) it is considered possible to randomise and collect data on 88 patients in 3 years time. After one year, the inclusion rate will be assessed. If accrual is too slow, additional centres will be invited to participate.

### Post-randomisation treatment protocol

All patients receive oral nutrition. If this is not tolerated, a nasojejunal feeding tube is introduced and enteral feeding is started [19]. If gastrointestinal feeding is contra-indicated, the patient will receive parenteral nutrition. Antibiotic treatment, Imipenem-cilastatin therapy 500 mg 3 times daily, is started in all patients with a maximum duration of fourteen days. Antibiotic treatment is switched based on blood cultures and culture from material collected during drainage and/or surgical procedures. If cultures remain negative antibiotic treatment is stopped. Selective decontamination of the digestive tract is allowed.

### Group A: minimally invasive 'step-up approach'

#### Step 1: Drainage

A percutaneous drain (at least 12 French) is placed in the (peri-)pancreatic collection. Multiple drains may be indicated in case of large or multiple collections. The preferred route is through the left retroperitoneum. If this is not possible a transperitoneal route is chosen. A right retroperitoneal route is allowed when it can be safely applied. If this is also not possible, an endoscopic transgastric drainage is performed with two 10 French drains (10 French being the current maximal drain size for endoscopic procedures) including a nasocystic drain for flushing. No continuous lavage system is installed. Drains are kept open by flushing with 50 ml saline once every 8-hrs shift by nursing staff. Extra saline may be used depending on the aspect of the return-fluid, the size of the collections on CECT, communication between drains and connections between collections. If more than 200 ml saline is used daily a drain fluid-balance chart is kept.

If there is no clinical improvement 72 hours after drain placement, this is considered a failure and a CECT is made to check the position of the drain. "Clinical improvement" is defined as: improved function of at least two organ systems (circulatory, pulmonary, renal) within 72 hours, or at least 10% improvement of two out of three parameters: leucocytes/temperature/CRP. If during the repeat CECT the position of the drain is adequate and no additional drainable collections are seen, the patient is taken to the operating room (step two). If the position of the drain is *in*adequate a second drain is placed in the collection.

Seventy-two hours after a second drainage-procedure the patient is again evaluated. In case of improvement, treatment is conservative; otherwise the patient will be taken to the operating room (step two). If after drainage, at any moment in time, a deterioration of at least two organ systems (circulatory, pulmonary, renal), or at least 10% deterioration of two out of three parameters: leucocytes/temperature/CRP occurs, step two is taken. Deterioration (of these parameters) by other infectious causes (e.g. an urinary tract infection) should be excluded.

#### Step two: videoscopic assisted retroperitoneal debridement

**Figure 2:**
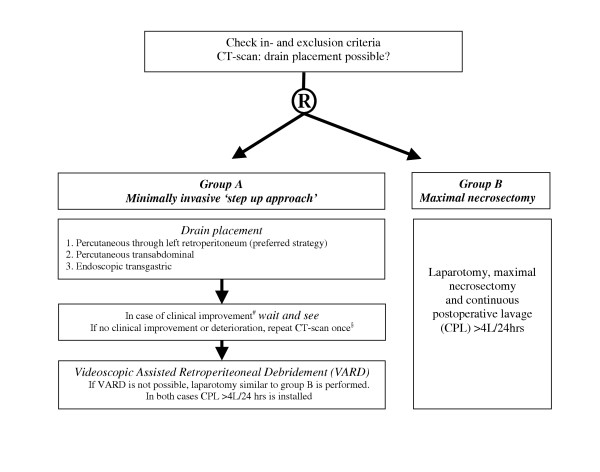
PANTER flowchart. ^® ^= randomisation. ^#^In the intensive care: within 72 hours, improved function of at least two organ systems (renal/respiratory/cardiovascular) **or **in the ward: within 72 hours, improvement of two of these three parameters: leucocytes/temperature/CRP. The worst parameter is used. ^§^No clinical improvement within 72 hours after drainage is considered failure: repeat the CT-scan once to check position of the drains. If the position is adequate, and no additional drainable collections are seen, proceed to surgery, if drain position is inadequate: repeat CT-guided percutaneous drainage (or endoscopic transgastric drainage). If after the second drainage there is no clinical improvement within 72 hours, proceed to surgery. If repeat-drainage is not possible, patients proceed to surgery.

The percutaneous retroperitoneal drain is used for videoscopic assisted retroperitoneal debridement (VARD). VARD has been used in several of the participating centres since 2000. The technique was recently described by Horvath *et al*[11]. See figure 2.

1. The patient is placed in a supine position with the left side elevated.

2. The left flank and the entire abdomen are prepared and draped.

3. Based on the position of the drain a 5 cm sub- or intercostal incision is made in the left flank.

4. With digital exploration the retroperitoneal drain is followed into the collection.

5. The collection is opened and necrosectomy performed with ring forceps and suction device.

6. A long 10 mm trocar and a 10 mm long zero degree videoscope (laparoscope) are inserted through the incision in the retroperitoneum.

7. With the videoscope the cavity is inspected and remaining, loosely adherent, necrosis is removed with a laparoscopic grasper or ring forceps. Note: it is not considered mandatory to remove all necrotic material.

8. Two large bore surgical drains are placed in the collection via the incision and the skin is closed between the drains.

If VARD is technically not feasible, laparotomy by bilateral subcostal incisions is performed as in Group B. Outcome of these patients will be analysed in group A according to the intention-to-treat principles.

### Group B: maximal necrosectomy by laparotomy

Laparotomy is performed with a bilateral subcostal incision. The lesser sac is entered through the omentum and carefully inspected. Blunt debridement of all necrotic tissue is performed. Two large bore drainage tubes are inserted through separate incisions with their tips in the lesser sac and necrotic cavities, the entrance to the lesser sac is carefully closed to create a contained space for CPL [20]. In case of diffuse bleeding, it is advisable to perform packing with gauzes that are to be removed the next day. Consequently, the lavage system is then installed on the first postoperative day.

Both in group A and in group B, necrotic material is collected during necrosectomy for culture and the total amount of necrotic tissue collected is weighed and photographed.

### Postoperative management

The postoperative management is similar in both groups. Continuous postoperative lavage with normal saline or peritoneal dialysis (CAPD) fluid is started. On the third postoperative day, the lavage should amount to at least 10 litres per 24 hours. CECT is performed one week after every drain placement and surgical intervention. Catheters are removed if collapse of the cavity is shown on CECT with contrast through the drainage system (or: on a sinogram of the cavity), and daily production of clear fluids has decreased below 50 ml/24 hours. Patients can be discharged with catheters in place and continue irrigation at home. Time to final removal of the drains will be recorded.

#### Indication for re-intervention

Re-intervention is performed 'on-demand'. Planned re-intervention is only performed in case 'packing materials' have been left in-situ during the first procedure. A re-intervention for removal of necrotic remnants is, whenever technically feasible, performed in accordance with the strategy the patient was initially assigned to. However, a pancreatic abscess (a collection consisting only of pus without necrosis) is treated by percutaneous drainage only.

#### Data collection

Data are collected via a secured Internet module which was specifically designed for the PANTER trial (Julius Center for primary care and health sciences, UMC Utrecht, the Netherlands). Data are entered online but are not stored on the Internet. Sequential Organ Dysfunction Scores (SOFA) are noted at randomisation [21,22]. The SOFA score, major morbidity (Table 1) and other complications are registered every second day for the first month and twice weekly thereafter until discharge. Furthermore at any (re-)intervention the SOFA score, procedure-related data (length of procedure, blood-loss) and procedure-related complications are scored.

### Data monitoring

There will be regular contact (by telephone, e-mail and site-visits) between the study coordinators and participating centres. Two study nurses will monitor the entered data. Twice a year a minimum of 10% of the data, including all end points, will be verified (double-checked) with source data at the study site by an independent monitor.

### Follow-up

Patients are observed during their hospital stay. Follow-up visits are planned after 3 and 6 months. For the primary endpoint, follow-up is completed at 3 months and for the secondary endpoints at 6 months after discharge, with a physical examination to exclude incisional hernia and ultrasonography to exclude a pancreatic pseudocyst.

## Discussion

A recent Dutch retrospective multicenter study showed that laparotomy with CPL is the current standard for treatment of infected necrotizing pancreatitis as it is the technique most frequently used in the Netherlands with consistently and relatively good results [8]. In this series, mortality for the 'open-abdomen-strategy' was 70% (intention-to-treat analysis) which is unacceptably high. The 'experimental arm' in PANTER is a minimally invasive 'step-up approach'. Notably, in necrotizing pancreatitis a minimally invasive approach does not only aim at minimising surgical stress, it is also part of a different treatment concept. We hypothesize that it is not necessary to remove all necrotic tissue in order to successfully treat patients with infected necrotizing pancreatitis. By performing drainage of 'infected fluid under pressure' the clinical condition may improve and the necrotic tissue may successfully be dealt with by the patient's immune system. It is therefore not the goal of drainage to remove (peri-)pancreatic necrosis but merely the infected fluid.

The second step of the minimally invasive 'step-up approach', 'VARD', is only performed if the first step fails. VARD combines endoscopic 'drain-tract' necrosectomy as first described by Carter *et al*[9]. and an open retroperitoneal approach. In our opinion VARD has the advantage of endoscopic necrosectomy as it is minimally invasive. However, VARD lacks the disadvantages of being technically demanding and time consuming. In VARD the relatively small 5 cm incision greatly facilitates the 'debulking' part. This will not only reduce the operating time but also potentially reduce the number of procedures needed to remove the necrotic tissue.

It has been argued that minimally invasive procedures are only feasible in a small subgroup of necrotizing pancreatitis patients. Our group recently performed a feasibility study re-evaluating CT scans of 80 patients operated upon for (suspected) infected necrotizing pancreatitis and found that in the vast majority of patients PCD is technically feasible and that in the majority of patients VARD is possible [23].

Currently, there are no randomised controlled trials comparing surgical techniques in necrotizing pancreatitis most likely due to low patient volumes, the heterogeneity of the disease and the highly individualized approach. The Dutch Acute Pancreatitis Study Group was founded to resolve several of these problems [24]. Since 2004, 15 of the 20 centres participating in PANTER are also participating in PROPATRIA, a placebo-controlled trial of probiotic prophylaxis in predicted severe acute pancreatitis [25]. Patients who initially are included in PROPATRIA are screened for eligibility in PANTER. Once a patient develops infected necrotizing pancreatitis the outcome for PROPATRIA is reached and he/she can be randomised for PANTER. With this already ongoing multicenter cooperation the logistical and organisational problems are expected to be kept at a minimum.

The sequential analysis of the primary outcome enables us to stop the trial as soon as sufficient evidence has accumulated regarding both clinically relevant and statistically significant treatment difference. Compared to a conventional fixed sample size sequential analysis on average requires less patients to show an expected effect if it truly exists [18]. This can be advantageous when the disorder is as rare as infected necrotizing pancreatitis. Moreover, as soon as the predefined threshold is passed randomisation may seize, thus preventing further patients to be subjected to an inferior treatment. If the presumed effect does not exist, based on conventional sample size calculations, 88 patients will be randomised.

A possible disadvantage of the present study concept is the large number of centres participating. In the Netherlands centralisation has not reached a level that all patients are referred to tertiary centres [8,26]. However, the 20 hospitals participating are amongst the largest of the 101 Dutch hospitals and all have experienced gastrointestinal surgeons, gastroenterologists and radiologists, including for example interventional radiology facilities to treat imminent bleeding. Furthermore, since in the Netherlands distances between any participating hospital and the nearest university medical centre is always within 10–100 kilometres, referral of patients is generally accepted and easy. A second possible disadvantage is the exclusion criteria 'previous placement of percutaneous drains'. This may apply to a considerable number of patients. The steering committee will repeatedly address this issue in meetings with referring physicians. Finally, in a trial with a rare disease such as infected necrotizing pancreatitis accrual is expected to be difficult. If after the first year less than 85% of the expected patients are recruited the study group will invite (inter-)national centres to join PANTER.

## Conclusion

PANTER is a randomised controlled multicenter trial set out to reveal a reduction in major morbidity by introducing a minimally invasive 'step-up approach' instead of maximal necrosectomy by laparotomy in patients with (suspected) infected necrotizing pancreatitis. Results are expected by 2008.

## Competing interests

The author(s) declare that they have no competing interests.

## Authors' contributions

MGHB drafted the manuscript

HCVS and HGG co-authored the writing of the manuscript

MGHB, HCVS, VBN, MAB, TLB, EB, CHCD, CHJVE, HVG, HSH, JSL, MSVL, RJP, BVR, AFMS, DJG, IVDT, BJMW and HGG participated in the design of the study during several meetings of the Dutch Acute Pancreatitis Study Group

MGHB, IVDT and EB performed the sample size calculations.

All authors edited the manuscript and read and approved the final manuscript.

## Appendix

### PANTER committee members

#### Steering Committee

HG Gooszen (chairman), HC van Santvoort (principal investigator), MGH Besselink (trial infrastructure), E Buskens, MS van Leeuwen, UMC Utrecht; MA Boermeester, JS Lameris, AMC Amsterdam; CHJ van Eijck, ErasmusMC Rotterdam; TL Bollen, B van Ramshorst, St. Antonius Hospital Nieuwegein; RJ Ploeg, HS Hofker, UMC Groningen; H van Goor, Radboud University Nijmegen MC; CHC Dejong, University Hospital Maastricht; AFM Schaapherder, Leiden UMC.

#### Monitoring Committee

FL Moll (Chairman), KG Moons, M Samsom, M Prokop, UMC Utrecht; PB Soeters, University Hospital Maastricht.

### Key staff at coordinating centre

HC van Santvoort (principal investigator), MGH Besselink (trial infrastructure), VJM Zeguers (trial research nurse), AJ Roeterdink (trial research nurse), GA Cirkel (investigator), J Oors (auditor), E Buskens (epidemiologist), RER Veen, JW Maaskant (internet randomisation and data entry), HG Gooszen (supervisor), University Medical Center Utrecht.

### Clinical centres and investigators

The last investigator per hospital is the local principal investigator. All investigators are from departments of Surgery, unless specified (G) departments of Gastroenterology or (R) departments of Radiology. University Hospital Groningen: HS Hofker, DM van Dullemen (G), EJ van der Jagt (R), RJ Ploeg; UMC St. Radboud Nijmegen: JBMJ Janssen (G), SP Strijk (R), H van Goor; University Hospital Maastricht: JW Greve, W Hameeteman (G), R Vliegen (R), CHC Dejong; Erasmus Medical Center Rotterdam: EJ Kuipers (G), JJ Hermans (R), JF Lange, CHJ van Eijck; Academic Medical Center Amsterdam: MJ Bruno (G), JS Lameris (R), DJ Gouma, MA Boermeester; Leiden University Medical Center: A Haasnoot, AFM Schaapherder; Vrije Universiteit Medical Center Amsterdam: CJ Mulder (G), MA Cuesta; Canisius Wilhelmina Hospital Nijmegen: AC Tan (G), PH Haarbrink (R), AHM Molenaar (R), C Rosman; Medical Center Rijnmond Zuid: E van der Harst; Gelderse Vallei Hospital Ede: PhM Kruyt, BJM Witteman (G); Meander Medical Center Amerfoort: MA Brink (G), BGF Heggelman (R), ECJ Consten; Hospital Arnhem: PJ Wahab (G), EJ Spillenaar Bilgen; Reinier de Graaf Group Delft: LPS Stassen, CJM Bolwerk (G), TM Karsten; Medical Spectrum Twente Enschede, JJPG Gerritsen, JJ Kolkman (G), AB Huisman (R), RJ de Wit; Gelre Hospitals Apeldoorn: EJ Hesselink; Medical Center Alkmaar: HARE Tuynman (G), BM Wiarda (R), APJ Houdijk; Medical Center Leeuwarden: P Spoelstra (G), JA Dol (R), JPEN Pierie; St. Elisabeth Hospital Tilburg: AWM van Milligen de Wit (G), CJHM van Laarhoven, St. Antonius Hospital Nieuwegein: R Timmer (G), BLAM Weusten (G), TL Bollen (R), B van Ramshorst; University Medical Center Utrecht: HC van Santvoort, MGH Besselink, E Buskens, MP Schwartz (G), MS van Leeuwen (R), HG Gooszen

## Pre-publication history

The pre-publication history for this paper can be accessed here:



## Figures and Tables

**Table 1:** Primary outcome of the PANTER trial; complications after first intervention

Mortality
New-onset multi organ failure or systemic complications ^#^
Enteric fistula (either small or large bowel)
Perforation of a visceral organ requiring intervention
Bleeding requiring intervention*

^# ^New onset failure of 2 or more organ systems or systemic complications as listed in Table 2, occurring at the same moment in time.

*Surgical, endoscopic or radiological

**Table 2:** Definitions of organ failure and systemic complications

Organ Failure	Circulatory	Systolic blood pressure less than 90 mm hg or need for catecholamine support	
	Pulmonary	PaO_2 _60 mm Hg or less or need for mechanical ventilation	
	Renal	creatinine level greater than 177 umol/L after rehydration or or need for hemofiltration or hemodialysis	

Systemic complications	Gastrointestinal bleeding	more than 500 ml/24 hours	
	Disseminated intravascular coagulation	platelets <100 × 10^9^/L	
	Severe metabolic disturbance	calcium level <1.87 mmol/L	

## References

[B1] Bradley EL (1993). A clinically based classification system for acute pancreatitis. Summary of the International Symposium on Acute Pancreatitis, Atlanta, Ga, September 11 through 13, 1992. Arch Surg.

[B2] (2005). UK guidelines for the management of acute pancreatitis. Gut.

[B3] Toouli J, Brooke-Smith M, Bassi C, Carr-Locke D, Telford J, Freeny P (2002). Guidelines for the management of acute pancreatitis. J Gastroenterol Hepatol.

[B4] Uhl W, Warshaw A, Imrie C, Bassi C, McKay CJ, Lankisch PG (2002). IAP Guidelines for the Surgical Management of Acute Pancreatitis. Pancreatology.

[B5] Mier J, Luque-de León E, Castillo A, Robledo F, Blanco R (1997). Early versus late necrosectomy in severe necrotizing pancreatitis. Am J Surg.

[B6] Nieuwenhuijs VB, Besselink MG, van Minnen LP, Gooszen HG (2003). Surgical management of acute necrotizing pancreatitis: a 13-year experience and a systematic review. Scand J Gastroenterol Suppl.

[B7] Werner J, Feuerbach S, Uhl W, Buchler MW (2005). Management of acute pancreatitis: from surgery to interventional intensive care. Gut.

[B8] Besselink MGH, De Bruijn MT, Rutten JP, Boermeester MA, Hofker HS, Gooszen HG, Dutch Acute Pancreatitis Study Group (2006). Surgical intervention in patients with necrotizing pancreatitis. Br J Surg.

[B9] Carter CR, McKay CJ, Imrie CW (2000). Percutaneous necrosectomy and sinus tract endoscopy in the management of infected pancreatic necrosis: an initial experience. Ann Surg.

[B10] Connor S, Ghaneh P, Raraty M, Sutton R, Rosso E, Garvey CJ (2003). Minimally invasive retroperitoneal pancreatic necrosectomy. Dig Surg.

[B11] Horvath KD, Kao LS, Wherry KL, Pellegrini CA, Sinanan MN (2001). A technique for laparoscopic-assisted percutaneous drainage of infected pancreatic necrosis and pancreatic abscess. Surg Endosc.

[B12] Echenique AM, Sleeman D, Yrizarry J, Scagnelli T, Guerra-JJ J, Casillas VJ (1998). Percutaneous catheter-directed debridement of infected pancreatic necrosis: results in 20 patients. J Vasc Interv Radiol.

[B13] Freeny PC, Hauptmann E, Althaus SJ, Traverso LW, Sinanan M (1998). Percutaneous CT-guided catheter drainage of infected acute necrotizing pancreatitis: techniques and results. Am J Roentgenol.

[B14] Gouzi JL, Bloom E, Julio C, Labbe F, Sans N, el Rassi Z (1999). [Percutaneous drainage of infected pancreatic necrosis: an alternative to surgery]. Drainage percutane des necroses pancreatiques infectees: alternative a la chirurgie. Chirurgie.

[B15] Baron TH, Thaggard WG, Morgan DE, Stanley RJ (1996). Endoscopic therapy for organized pancreatic necrosis. Gastroenterology.

[B16] Seewald S, Groth S, Omar S, Imazu H, Seitz U, de Weerth A (2005). Aggressive endoscopic therapy for pancreatic necrosis and pancreatic abscess: a new safe and effective treatment algorithm (videos). Gastrointest Endosc.

[B17] Mueller PR (1998). Percutaneous drainage of pancreatic necrosis: is it ecstasy or agony?. AJR Am J Roentgenol.

[B18] Whitehead J (1997). The design and analysis of sequential clinical trials.

[B19] Marik PE, Zaloga GP (2004). Meta-analysis of parenteral nutrition versus enteral nutrition in patients with acute pancreatitis. BMJ.

[B20] Beger HG, Buchler M, Bittner R, Block S, Nevalainen T, Roscher R (1988). Necrosectomy and postoperative local lavage in necrotizing pancreatitis. Br J Surg.

[B21] Vincent JL, de Mendonca A, Cantraine F, Moreno R, Takala J, Suter PM (1998). Use of the SOFA score to assess the incidence of organ dysfunction/failure in intensive care units: results of a multicenter, prospective study. Working group on "sepsis-related problems" of the European Society of Intensive Care Medicine. Crit Care Med.

[B22] Vincent JL, Moreno R, Takala J, Willatts S, de Mendonca A, Bruining H (1996). The SOFA (Sepsis-related Organ Failure Assessment) score to describe organ dysfunction/failure. On behalf of the Working Group on Sepsis-Related Problems of the European Society of Intensive Care Medicine. Intensive Care Med.

[B23] Besselink MG, Van Santvoort H, Bollen TL, Van Leeuwen MS, Hofker S, Boermeester MA (2005). Minimally Invasive Approach in Acute Necrotising Pancreatitis: a Strategy for a Selected Subgroup or a Potential Benefit for All? Dutch Acute Pancreatitis Study Group. Gut.

[B24] Besselink MG, Bollen TL, Boermeester MA, Van Ramshorst B, van Leeuwen MS, Gooszen HG (2005). [Timing and choice of intervention in necrotising pancreatitis]. Ned Tijdschr Geneeskd.

[B25] Besselink MGH, Timmerman HM, Buskens E, Nieuwenhuijs VB, Akkermans LMA, Gooszen HG (2004). Probiotic prophylaxis in patients with predicted severe acute pancreatitis (PROPATRIA): design and rationale of a blinded, placebo-controlled randomised controlled trial. Dutch Acute Pancreatitis Study Group [ISRCTN38327949]. BMC Surgery.

[B26] van Heek NT, Kuhlmann KF, Scholten RJ, de Castro SM, Busch OR, Van Gulik TM (2005). Hospital volume and mortality after pancreatic resection: a systematic review and an evaluation of intervention in the Netherlands. Ann Surg.

